# Use of probiotics in clinical practice with special reference to diarrheal diseases: A position statement of the Malaysian Society of Gastroenterology and Hepatology

**DOI:** 10.1002/jgh3.12469

**Published:** 2020-12-12

**Authors:** Yeong‐Yeh Lee, Alex H‐R Leow, Pei‐Fan Chai, Raja Affendi Raja Ali, Way‐Seah Lee, Khean‐Lee Goh

**Affiliations:** ^1^ Department of Medicine, School of Medical Sciences Universiti Sains Malaysia Kota Bharu Malaysia; ^2^ Gut Research Group, Faculty of Medicine Universiti Kebangsaan Malaysia Kuala Lumpur Malaysia; ^3^ Gastroenterology Unit, Department of Medicine, Faculty of Medicine Universiti Kebangsaan Malaysia Jalan Universiti Kuala Lumpur Malaysia; ^4^ University of Malaya Medical Centre Jalan Universiti Kuala Lumpur Malaysia; ^5^ Endoscopy Centre Pantai Hospital Kuala Lumpur Malaysia; ^6^ Department of Paediatrics, Faculty of Medicine University of Malaya Kuala Lumpur Malaysia; ^7^ Department of Medicine, Faculty of Medicine University of Malaya Kuala Lumpur Malaysia

**Keywords:** cellular imaging, dipolar dye, fluorescent dye, nanoprobe, near‐infrared emitting

## Abstract

Probiotics comprise a large group of microorganisms, which have different properties and thus confer different benefits. The use of probiotics has shown promising results in the management of diarrheal diseases. While the availability of probiotic products has flourished in the marketplace, there is limited guidance on the selection of probiotics for clinical use. This position paper is aimed at informing clinicians about the proper selection criteria of probiotics based on current evidence on strain‐specific efficacy and safety for the management of diarrheal diseases. Members of the working group discussed issues on probiotic use in clinical practice, which were then drafted into statements. Literature to support or refute the statements were gathered through a search of medical literature from 2011 to 2020. Recommendations were formulated based on the drafted statements and evidence gathered, revised as necessary, and finalized upon agreement of all members. Twelve statements and recommendations were developed covering the areas of quality control in the manufacturing of probiotics, criteria for selection of probiotics, and established evidence for use of probiotics in diarrheal diseases in adults and children. Recommendations for the use of specific probiotic strains in clinical practice were categorized as proven and probable efficacy based on strength of evidence. Robust evidence is available to support the use of probiotics for diarrheal diseases in clinical practice. Based on the results obtained, we strongly advocate the careful evaluation of products, including manufacturing practices, strain‐specific evidence, and contraindications for at‐risk populations when choosing probiotics for use in clinical practice.

## Graphical abstract

This is a position statement aimed at informing clinicians about the proper selection criteria of probiotics based on current evidence on strain‐specific efficacy and safety for management of diarrheal diseases.

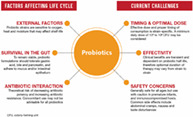

## Introduction

Various evidence supports the role of gut microbiota in human health and disease. Among these are the ability of the gut microbiota to modulate insulin sensitivity, gut inflammation, lipid metabolism and cardiovascular risk, development of obesity, and protection against some infections.^1^ Dysbiosis or microbial imbalance may occur due to several factors, including diet, infection, disease (e.g. irritable bowel syndrome (IBS), inflammatory bowel disease (IBD), type 1 and 2 diabetes, atopic eczema, and obesity), and drugs (e.g. antibiotics).^1^, ^2^ Probiotics and prebiotics are among the most well‐established interventions in modulating the gut microbiota for health benefits.^1^ Specifically, probiotics are able to restore gastrointestinal (GI) homeostasis via the actions of beneficial microbes, thus conferring therapeutic benefits in disease states.^2^

Recently, a group of Malaysian experts in gut health developed a position paper on selected key issues surrounding gut microbiota in early life, the role of gut microbiota in gut health and diseases, and the group's stand on the general role of probiotics and prebiotics.^3^ With increasing evidence on the benefits of probiotics, interest and awareness of its general use among consumers have grown by leaps and bounds in recent times. In parallel, there has been an exponential growth of probiotic products present in the market, most of which are available as over‐the‐counter health supplements or added to food products.^4^ These products are available in a variety of preparations, such as mono‐ or mixed cultures of live bacteria or yeasts, and are formulated as pills, sprays, liquids, suspensions, capsules, and a host of other forms.^4^

In the context of clinical use, at the regional level, a multidisciplinary panel of clinician scientists and clinicians from five Southeast Asian countries, including Malaysia, has developed a set of statements to guide health‐care professionals in evaluating the efficacy and safety of probiotic therapy.^5^ There is, however, limited guidance from local medical bodies on the use of probiotics for clinical conditions. Available clinical guidelines in Malaysia are limited to providing brief and general recommendations on the use of probiotics for the management of acute diarrhea,^6^, ^7^ necrotizing enterocolitis,^8^ and prevention of eczema.^9^ There is no clear guidance provided on the selection of probiotics according to strain specifications, which is a critical factor in ensuring the efficacy of probiotics. A clear gap is therefore present in the efforts to promote the appropriate and judicious use of probiotics in clinical practice.

This position paper is intended to serve as a practical guidance for health practitioners in Malaysia on the use of probiotics in the management of diarrheal diseases; however, it is also relevant to clinicians outside Malaysia. The recommendations are aimed at informing clinicians about the proper selection criteria of probiotics based on current evidence on strain‐specific efficacy and safety for the management of diarrheal diseases. Panel statements are listed in Table 1.

**Table 1:** Panel statements on the use of probiotics in clinical practice

Quality control in manufacturing of probiotics
Statement 1: Probiotic strains should be characterized and deposited into an internationally recognized culture collection Statement 2: Manufacturing process should be optimized as a guarantee of quality Statement 3: Probiotics registered as drugs, in general, conform to higher standards compared to food supplements Statement 4: Efficacy and safety of probiotic strains should be confirmed by well‐conducted randomized controlled clinical trials
Criteria for selection of probiotics
Statement 5: Strain specificity is an important factor that determines the efficacy of probiotics in diarrhea Statement 6: While generally regarded as safe, probiotics should be judiciously used, especially in at‐risk populations Statement 7: The properties of probiotics are different between those that contain yeast and bacteria Statement 8: It is unclear if a single‐strain or multistrain probiotic is more effective in diarrhea
Established evidence for use of probiotics
Statement 9: Probiotics are effective and safe in acute infectious diarrhea Statement 10: Probiotics are effective and safe in antibiotic‐associated diarrhea, including *Clostridium difficile*‐associated diarrhea Statement 11: Probiotics are effective and safe in preventing traveler's diarrhea Statement 12: Probiotics are indicated in irritable bowel syndrome for adults

## Methods

Members of the working group include adult and pediatric gastroenterologists who are key opinion leaders and researchers in the field of probiotics in Malaysia. Five members were invited to the first meeting to discuss issues on probiotic use in clinical practice based on experience and current literature from Malaysia and beyond. Points raised during the meeting were then drafted into statements by two members of the group. The statements comprised general information pertaining to probiotic regulation and use (statements 1–8) and specific guidance on selected clinical indications (statements 9–12). These two types of panel statements were judged to be the most relevant and beneficial to health‐care professionals in their clinical practice based on the current assessment of knowledge gaps on probiotics among health‐care professionals.

Literature to support or refute the statements were gathered based on evidence published from Malaysia, and if not available, papers outside of Malaysia were included. This was done through a search of medical literature in the English language from 2011 to 2020 using PubMed, Scopus, and Google Scholar databases. Search terms included: probiotics, diarrheal diseases, guidelines, randomized controlled trials (RCTs), systematic review, and meta‐analyses. Recommendations were formulated based on the drafted statements and evidence gathered, after which all group members were asked to provide their feedback independently. The statements and recommendations were further revised based on the feedback and justifications received from all members. A unanimous consensus was achieved among all members on the final statements and recommendations presented here.

## Results

### *Quality control in manufacturing of probiotics*

#### *Statement 1: Probiotic strains should be characterized and deposited into an internationally recognized culture collection*

Each probiotic strain is typically described by its genus, species, and a strain designation.^10^ Strain designations are usually chosen by researchers or marketers of a specific strain and do not follow any specific conventions.^10^ For example, in the probiotic *Lactobacillus rhamnosus* GG, *Lactobacillus* is the genus, *rhamnosus* is the species, and GG is the strain designation.^10^ The three components of a probiotic strain's name are essential in identifying the specific health benefits and safety assessments associated with that strain.^10^ Health benefits of one strain may not be extrapolated to other strains, even within the same species. Conversely, several probiotic mechanisms responsible for certain benefits may be commonly shared among most strains of a larger taxonomic group.^10^

Probiotic cultures should be deposited into an internationally recognized culture collection (e.g. American Type Culture Collection [ATCC] or Collection Nationale de Cultures de Microorganismes [CNCM]) to provide a long‐term source of reference material for the confirmation of genetic stability and for documentation of its strain‐specific mechanism of action. The Budapest Treaty on the International Recognition of the Deposit of Microorganisms for the Purpose of Patent Procedure 1977 states that the deposit of microorganisms with any international depository authority, such as ATCC and CNCM, is recognized.^11^ There are at least 47 such authorities recognized by the Treaty, including centers in Korea, China, India, and Japan in Asia.^11^

**Panel recommendation 1**

When choosing a probiotic product, ensure that the genus, species, and strain designation of each strain included in the product are listed in the product label or insert.

#### *Statement 2: Manufacturing process should be optimized as a guarantee of quality*

**Figure 1:**
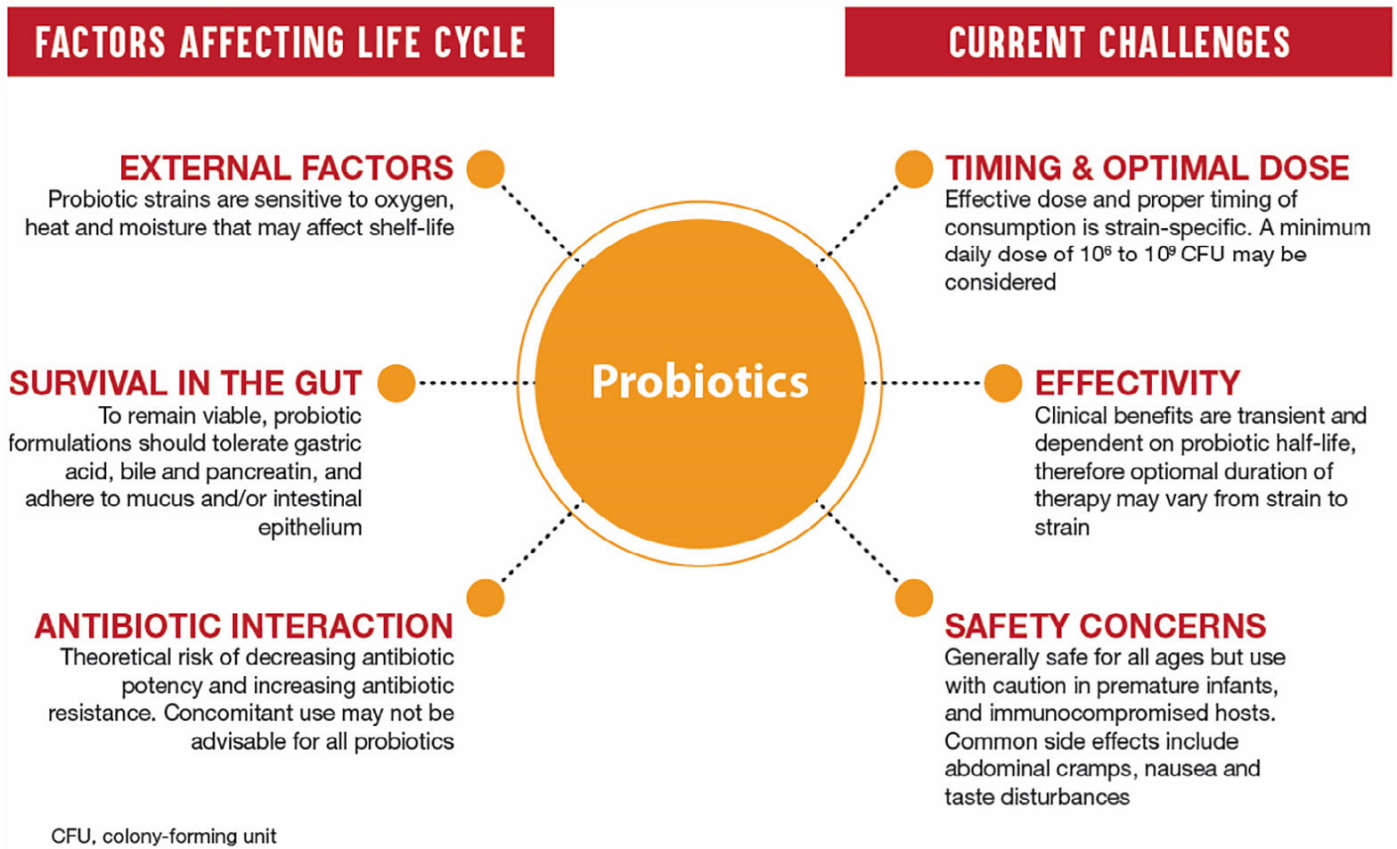
Viability is critical to efficacy of probiotics and is dependent on external factors, ability to survive in the gut environment, and interaction with antimicrobial agents. Other challenges in ensuring probiotic efficacy are also highlighted.

Probiotics are defined as ‘live microorganisms which, when administered in adequate amounts, confer a health benefit on the host’.^12^ By this definition, it is made clear that, in order to be considered a probiotic, viability of the microorganisms is an essential requirement (Fig. 1). Stringent manufacturing processes must therefore be adhered to in ensuring the viability and stability of the microorganisms in the product.^4^ Bacterial gene expression is significantly affected by growth media and industrial processing parameters. Any modifications in the manufacturing unit, reagents, and protocol can change the immunological and biochemical profile of the final product.^4^ Therefore, the same probiotic strain from different sources may have significantly altered properties and may not result in the health benefits it is claimed to have. Studies that have tested commercial probiotic products reveal that among the quality issues seen are misidentification and therefore mislabeling of the incorporated probiotic strain in the product, low number of total viable counts, detection of undesired microflora, and poor functional properties that do not meet the criteria for probiotics.^13^ There is a need for greater control of the manufacturing process of probiotic products similar to the specific and stringent regulatory processes implemented for drug products to ensure the quality of probiotics that are marketed as dietary supplements.

**Panel recommendation 2**

Choose probiotic products produced by manufacturers that are able to provide assurance and evidence of stringent quality control measures employed in manufacturing processes.

#### *Statement 3: Probiotics registered as drugs, in general, conform to higher standards compared to food supplements*

Probiotic products available in the market are broadly categorized into three categories: (i) probiotic in food or supplement without health claim; (ii) probiotic in food or supplement with a specific health claim; and (iii) probiotic drug.^14^ The level of evidence required by regulatory agencies for approval of these products vary according to the claim made, but at the minimum, well‐conducted human studies are required to support the observed general beneficial effect.^14^

Probiotics registered as drugs have well‐defined indications and endorsement of their claims by the relevant authorities.^15^ The status of the drug also represents a guarantee of quality in terms of manufacturing processes. The Food and Drug Agency of the United States and the European Medicines Agency regulate certain probiotics as drugs depending on their intended use. Probiotic products in food or supplement without health claims do not need to provide evidence of efficacy for the specific strain included in the product.^14^

The marketplace carries an abundance of products labeled ‘probiotic’, but these products often do not meet minimum criteria, such as defined contents, appropriate viable count at end of shelf‐life, and suitable evidence of health and therapeutic benefits.^14^ Probiotic drugs are required to provide proof of delivery of a viable probiotic at an efficacious dose for the treatment or prevention of specific indications at the end of shelf‐life.^16^, ^17^

**Panel recommendation 3**

Probiotics that are registered as drugs are controlled with higher standards than those registered as supplements and are recommended. The next category of choice is probiotic supplement with a specific health claim. Probiotics in food with a specific health claim may be considered if its strain‐specific safety and efficacy have been established through RCTs. Probiotics in food or supplement without an evidence‐based health claim must not be used for clinical purposes.

#### *Statement 4: Efficacy and safety of probiotic strains should be confirmed by well‐conducted randomized controlled clinical trials*

The health effects conferred by probiotic preparations are strain‐specific and dose‐dependent. It is therefore prudent to recommend probiotics that have been tested for specific clinical end‐points when prescribing probiotics for therapeutic purposes.^15^ RCTs and systematic reviews or meta‐analyses of RCTs are gold standards when evaluating the effects of therapeutic and prophylactic interventions. Safety of probiotics should similarly be evaluated via rigorously conducted RCTs.^15^ Assessment of safety should consider factors including taxonomic classification, manufacturing controls excluding contamination, absence of association with infectivity or toxicity, absence of transferable antibiotic resistance genes, target population, administered dose, method of administration, and absence of allergenic material.^15^ Overall, hundreds of RCTs for various indications have shown that probiotics have an excellent safety record. However, caution should be exercised when recommending probiotics to vulnerable patient populations such as neonates born prematurely and severely ill or immunocompromised patients.^10^ In such cases, clinicians must carefully make a risk/benefit assessment prior to recommending use.^10^ Product manufacturers should be able to provide guidance regarding the type and extent of safety assessments that have been conducted on their products if further clarification is necessary.^10^

**Panel recommendation 4**

Choose probiotic products with safety and strain‐specific efficacy that have been assessed and confirmed in well‐conducted RCTs.

### *Criteria for the selection of probiotics*

#### *Statement 5: Strain specificity is an important factor that determines the efficacy of probiotics in diarrhea*

Strain specificity is a vital factor when selecting an appropriate probiotic product. Strong evidence is available for the efficacy of specific probiotics for diarrhea.^18^ Strain‐specific properties are attributed to the varying mechanisms of action against pathogens in different probiotic strains, including bacteriocins that directly kill or inhibit specific pathogens, destruction of pathogenic toxins, reinforcement of host cell integrity, resistance of pathogen colonization of host cells, restoration of dysbiosis of normal microflora, and the ability to upregulate or downregulate immune response.^18^, ^19^

Selecting the appropriate probiotic for diarrhea based on clinical evidence of strain‐specific efficacy, however, remains a challenging task for two reasons. First, shifting taxonomy of bacterial and fungal nomenclature over time and a lack of a global standard for naming strains make misidentification of strain types highly possible.^19^ Second, many published trials and meta‐analyses report probiotics either on a genus or species level and do not provide their specific strain designation.^19^

**Panel recommendation 5**

When selecting a probiotic product for the treatment or prevention of diarrhea, choose strains for which evidence of efficacy has been established through RCTs.

#### *Statement 6: While generally regarded as safe, probiotics should be judiciously used, especially in at‐risk populations*

Probiotic products that can be safely recommended for at‐risk populations may need more rigorous testing than what is sufficient for the general population. Examples of at‐risk populations are newborns including preterm neonates; patients requiring critical care; and people in an immunocompromised state, such as those with acquired immune deficiency syndrome or on immunosuppressant therapy.^20^, ^21^ Appropriate certificates of analysis that document standards must be available. Testing for specific pathogens or toxins might also be needed if the target group has a special vulnerability, for example, pregnant women exposed to *Listeria* sp. contamination in the probiotic products they consume are at risk of miscarriage. Physicians should also seek available guidance regarding standards for safe use for at‐risk populations. This guidance may be available from government authorities or organizations. In addition, products that are accompanied by rigorous third‐party verification reports on adherence to good manufacturing practices elicit more confidence regarding use for at‐risk populations. Finally, awareness of potential contraindications in immunocompromised patients or those undergoing long‐term corticosteroid treatment must guide decisions of using probiotics in this population. As a condition for product approval, regulatory agencies must ensure that manufacturers provide users with updated product information sheets with the latest information on risks for any adverse events, especially for vulnerable populations.

**Panel recommendation 6**

Precautions must be exercised when prescribing probiotics to vulnerable patient populations such as newborns including preterm neonates, patients requiring critical care and who are immunocompromised, and pregnant women.

#### *Statement 7: The properties of probiotics are different between those that contain yeast and bacteria*

Probiotic preparations include several different genera and species of bacteria and yeast. Commonly used bacteria are species of *Bifidobacterium* (*adolescentis*, *animalis*, *bifidum*, *breve*, and *longum*) and *Lactobacillus* (*acidophilus*, *casei*, *fermentum*, *gasseri*, *johnsonii*, *paracasei*, *plantarum*, *rhamnosus*, and *salivarius*), while the yeast *Saccharomyces boulardii* is also often chosen.^10^ Yeasts are eukaryotes, whereas bacteria are prokaryotes, which therefore indicates that they have different properties. Major differences between yeast and bacteria include cell size, cell wall material, optimal growth conditions including pH and temperature, resistance to antibiotics, and transmission of genetic material.^22^ These properties result in further implications on their probiotic activity.

**Panel recommendation 7**

Both yeast and bacteria may be used in probiotic products, with different properties present in the two types of products. Either type of product may be used depending on their strain‐specific evidence for various types of diarrheal disease.

#### *Statement 8: It is unclear if a single‐strain or multistrain probiotic is more effective in diarrhea*

Probiotic preparations are available either as single‐strain or multistrain products. An increasing tendency has been observed for commercial products to have multiple strains of microorganism, particularly products with a high number of different strains.^23^ The rationale behind this is that more strains imply a broader spectrum of efficacy and, potentially, additive or synergistic effects of the strains.^23^ However, there is currently no evidence to support any assumptions of superiority of multiple strains over single‐strain probiotics.^24^ Conversely, no evidence shows inferiority of multistrain probiotics over single‐strain products and/or antagonistic activity between strains in a preparation.^23^, ^24^ Further research is needed in this area before conclusive recommendations can be made over the benefits of either preparation. As emphasized earlier, the selection of any probiotic products should be guided by convincing strain‐specific evidence for the health claim made.

**Panel recommendation 8**

Multistrain probiotic efficacy in diarrhea should not be considered the cumulative effect of single‐strain probiotics. Always choose probiotic products based on available evidence of strain‐specific efficacy.

### *Probiotics in diarrhea*

Diarrheal diseases contribute to a significant burden of morbidity and mortality in both adults and children.^25^, ^26^ In children, the mainstay of management of acute diarrheal illness is the prevention of dehydration and electrolyte imbalance by oral rehydration solution, as well as the provision of adequate nutrition. Antibiotics can be considered under specific circumstances, such as the very young or those who are immunocompromised and are at risk of developing invasive complications. Other measures such as antidiarrheal drugs and probiotics are considered to play a complementary role.

There is extensive evidence indicating that dysbiosis can be implicated in the development of GI diseases, including diarrhea, IBS, and IBD.^27^ Dysbiosis is described as the state in which there is a loss of beneficial microbes, expansion of pathogenic microbes, and reduced diversity of microbes.^28^ In diarrhea and other GI diseases, this state may arise due to infections by pathogens such as rotavirus, *Clostridium difficile*, *Shigella*, and *Aeromonas*; environmental insults such as use of antibiotics, dietary habits, and geographic location; and genetics and age.^25^, ^26^, ^28^

The use of probiotics to restore the normal gut microbiota is an area of growing interest and in which numerous studies have been conducted. Specifically, clinical trials have been conducted to determine the efficacy of various probiotic products in the management of diarrheal diseases. The availability of sufficient evidence to indicate that probiotics are effective in the prevention or treatment of diarrheal diseases has led to probiotic products being considered an important therapeutic option in clinical practice.

In this section, we present evidence‐based proven and probable strain‐specific efficacy of probiotic products in specific types of diarrheal diseases (Table 2). ‘Proven efficacy’ is defined by the highest evidence from RCTs or meta‐analyses and would be compatible with Grading of Recommendations, Assessment, Development and Evaluations (GRADE) levels of moderate to high quality of evidence.^29^ ‘Probable efficacy’ is defined by evidence other than RCTs or meta‐analysis (e.g., open‐label and uncontrolled trials) and would be compatible with GRADE levels of very low to low quality of evidence.^29^

**Table 2:** Summary of recommendations for use of probiotics in diarrheal diseases

Condition	Proven efficacy	Probable efficacy
Acute infectious diarrhea		
Adults		
Treatment	*Lactobacillus rhamnosus* GG, *Saccharomyces boulardii* CNCM I‐745	*Lactobacillus paracasei* B21060, *Lactobacillus reuteri* DSM17938
Children		
Treatment	*L. rhamnosus* GG, *S. boulardii* CNCM I‐745	*Bacillus clausii*, *L. paracasei* B21060, *L. reuteri* DSM17938
Prevention		*L. rhamnosus* GG, *S. boulardii* CNCM I‐745
Antibiotic‐associated diarrhea		
Adults		
Treatment	*L. rhamnosus* GG, *S. boulardii* CNCM I‐745	
Prevention	*Lactobacillus bulgaricus*, *Lactobacillus casei* DN‐114001, *L. reuteri* ATCC 55730, *Streptococcus thermophilus*, and mixture of *Lactobacillus acidophilus* CL1285 + *L. casei* LBC80R + *L. rhamnosus* CLR2	
Children		
Treatment	*L. rhamnosus* GG, *S. boulardii* CNCM I‐745	
*Clostridium difficile*‐associated diarrhea		
Adults		
Prevention	Mixture of *L. acidophilus* CL1285 + *L. casei* LBC80R + *L. rhamnosus* CLR2, mixture of *Bifidobacterium bifidum* + *L. acidophilus*, *L. bulgaricus*, *L. casei* DN‐114001, *L. casei* LBC80R, *S. thermophilus*	
Children		
Prevention		*S. boulardii* CNCM I‐745
Traveler's diarrhea		
Adults		
Prevention	*S. boulardii* CNCM I‐745	
Irritable bowel syndrome		
Adults		
Treatment	*Bifidobacterium infantis* 35 624, *Escherichia coli* DSM17252, *Lactobacillus plantarum* 299v, *S. boulardii* CNCM I‐745	*B. bifidum* MIMBb75, VSL#3

#### *Statement 9: Probiotics are effective and safe in acute infectious diarrhea*

The use of probiotics for the treatment of acute infectious diarrhea has been extensively studied, especially in children. Probiotics, when used alongside rehydration therapy, appear to be safe and have clear beneficial effects in shortening the duration and reducing stool frequency, as well as reducing the duration of hospitalization in acute infectious diarrhea.

##### Proven efficacy

In adults and children, *L. rhamnosus* GG *and S. boulardii* CNCM I‐745 are recommended for the treatment of acute infectious diarrhea, including rotavirus diarrhea.^16^, ^26^, ^30^, ^31^, ^32^, ^33^

##### Probable efficacy

*Lactobacillus paracasei* B21060 and *L. reuteri* DSM17938 have also shown efficacy in the adult and pediatric populations, respectively.^16^, ^26^, ^30^, ^31^, ^33^ For the prevention of acute infectious diarrhea in children, *Bacillus clausii*, *L. rhamnosus* GG, and *S. boulardii* CNCM I‐745 have shown promising results.^31^, ^34^

#### *Statement 10: Probiotics are effective and safe in antibiotic‐associated diarrhea including* **C. difficile*‐associated diarrhea*

##### Proven efficacy

Robust evidence is available on the efficacy of probiotics in antibiotic‐associated diarrhea (AAD) and *C. difficile*‐associated diarrhea. *L. rhamnosus* GG and *S. boulardii* CNCM I‐745 are efficacious in adults and children with AAD.^16^, ^18^, ^26^, ^32^, ^33^, ^34^, ^35^ In addition, *L. bulgaricus*, *L. casei* DN‐114001 *L. reuteri* ATCC 55730, *Streptococcus thermophilus*, and a mixture of three strains of Lactobacillus spp. (*L. acidophilus* CL1285, *L. casei* LBC80R, and *L. rhamnosus* CLR2) have also shown efficacy in preventing AAD in adults.^16^ (Strains of *L. casei* and *L. paracasei* are widely used in food products and have been investigated in numerous clinical trials for a variety of health claims, including diarrhea. Some of these products have been found to contain adequate amounts of bacteria that may be considered within therapeutic ranges. This, however, is not indicative that all food products with claims of probiotic properties can be used for the treatment or prevention of diseases in clinical practice. Careful evaluation of claimed health benefits and supporting evidence must be performed before use in clinical practice can be advocated.) For the prevention of *C. difficile*‐associated diarrhea, Lactobacillus mixture, *L. bulgaricus*, *L. casei* DN‐114001, *L. casei* LBC80R, *S. thermophilus*, and a mixture of *B. bifidum and L. acidophilus* have shown effectiveness in adults.^16^, ^18^

##### Probable efficacy

*Saccharomyces boulardii* CNCM I‐745 may be considered for the prevention of *C. difficile*‐associated diarrhea in the pediatric population.^26^, ^32^, ^33^

#### *Statement 11: Probiotics are effective and safe in preventing traveler's diarrhea*

Probiotics have been found to significantly reduce the risk of traveler's diarrhea; however, the data supporting their use in preventing traveler's diarrhea is not consistently strong.^15^

##### Proven efficacy

Currently, only *S. boulardii* CNCM I‐745 appears to be a good candidate in preventing traveler's diarrhea among adults.^15^, ^18^, ^36^

The use of probiotics to prevent traveler's diarrhea among children is not currently recommended.^26^

#### *Statement 12: Probiotics are indicated in irritable bowel syndrome*

Multiple publications indicate that probiotics are an effective treatment for IBS.^37^, ^38^ Further evidence is, however, required on the role of individual species and strains. Most meta‐analyses of probiotics in IBS report its beneficial impact on global symptoms, abdominal pain, and flatulence.^39^

##### Proven efficacy

*Bifidobacterium infantis* 35 624, *E. coli* DSM17252, *L. plantarum* 299v, and *S. boulardii* CNCM I‐745 have resulted in improvements in global IBS symptoms, reduction of pain, and better quality‐of‐life scores in adults.^16^, ^18^, ^19^, ^40^

##### Probable efficacy

*B. bifidum* MIMBb75 and VSL#3 (*B. breve* BB02, *B. infantis* BI04, *B. lactis* BL03, *L. acidophilus* BA05, *L. helveticus* BD08, *L. paracasei* BP07, *L. plantarum* BP06, and *S. thermophilus* BT01) have also resulted in the reduction of global IBS symptoms and improved quality of life among adults with IBS and may be considered a treatment option.^16^, ^41^, ^42^, ^43^ In addition, a study conducted in two villages in Kelantan reported that *B. Infantis* M‐63 is probably effective in improving the mental well‐being of victims of major floods who developed IBS, potentially attributed to the restoration of microbial balance and the gut–brain axis.^44^

## Conclusion

**Figure 2:**
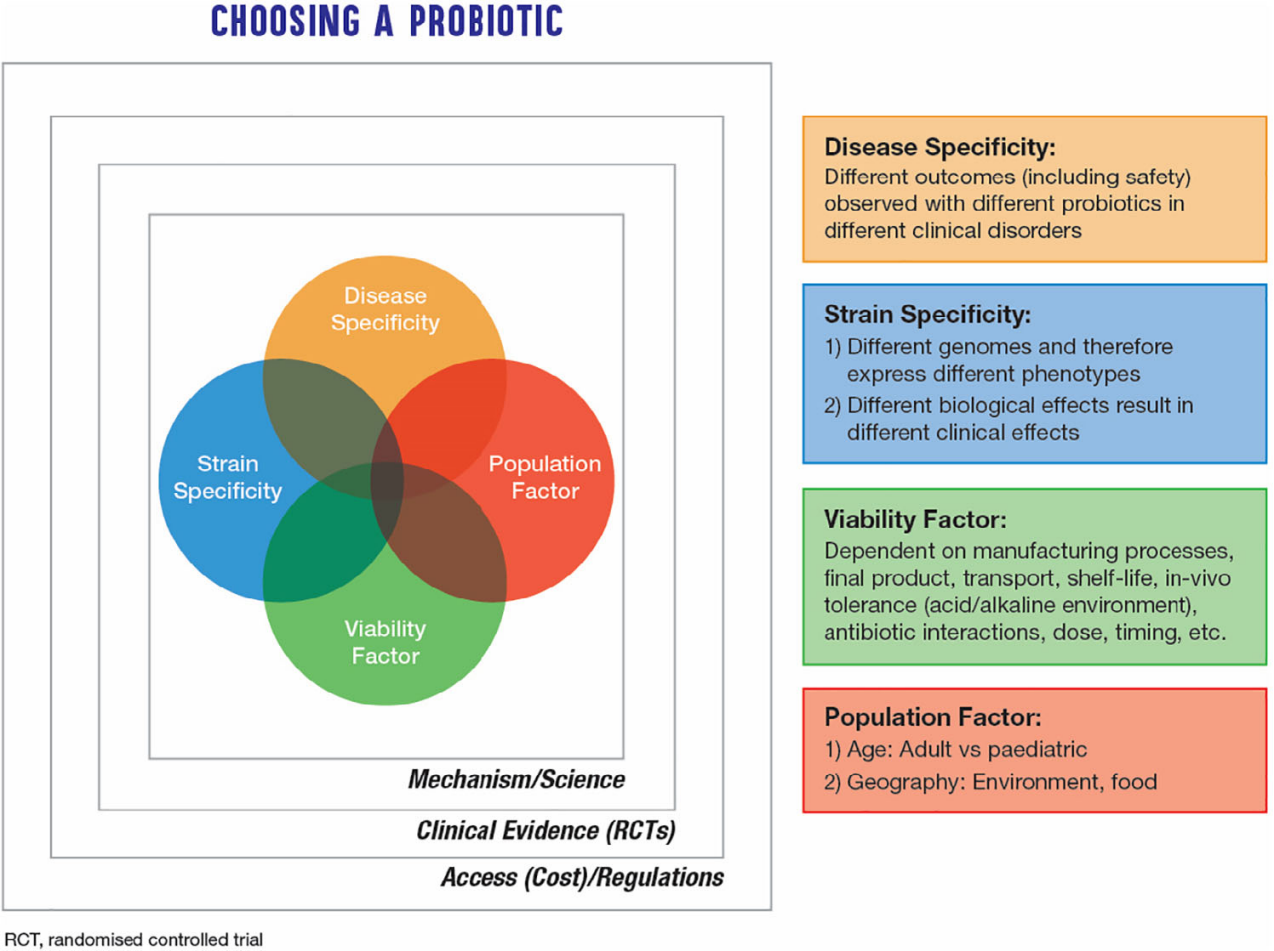
Considerations when choosing a probiotic for a clinical indication like diarrheal diseases.

This position paper presents evidence‐based guidelines on the use of probiotics in clinical practice for diarrheal diseases. The choice of probiotics for a clinical indication like diarrhea is dependent on a number of factors, and these are summarized in Figure 2. Clinicians may also use the checklist provided in Table 3 to guide their decisions when choosing a probiotic product in clinical practice. With the proliferation of probiotic products in the market, careful evaluation of the products must be conducted prior to recommending use in clinical practice, including manufacturing practices, health claims and strain‐specific evidence, and contraindications for at‐risk populations.

**Table 3:** Checklist for clinical decision regarding using probiotics for the management of diarrheal diseases^19^

Determine if purpose for use of probiotics is for disease treatment or prevention
Refer to evidence on efficacy from clinical trials to decided which probiotic strain or mixture of strains is appropriate for the specific condition being considered
Choose a probiotic product with appropriate strain or strains
Consider source of manufacture (quality control, production company)
Consider whether probiotic product complies with country's regulations
Decide on dosage and treatment according to evidence (adjunctive or main treatment)
Review contraindications
Review antibiotic–probiotic drug interactions
